# Supplementary material for: Pronounced gut microbiota signatures in patients with JAK2V617F-positive essential thrombocythemia
Source: Microbiol Spectr. 2023 Sep 11;11(5):e00662-23. doi: 10.1128/spectrum.00662-23 (PMC10581245; doi:10.1128/spectrum.00662-23)
Supplement: Table S1 and Fig. S1 — Table S1: baseline characteristics of patients with ET versus HC. Figure S1: differential abundance analysis of the gut microbiota in patients with ET according to mutations status compared with healthy controls. [file spectrum.00662-23-s0001.pdf]

## Supplementary tables and figures

Table S1. Baseline characteristics of patients with ET vs. healthy controls (HC)

| Characteristic | Group<br>*JAK2V617F* positive (A) | *JAK2V617F* negative (B) | HCs (C) | ***P value*** |
|---|---|---|---|---|
| **Number of patients** | 36 | 16 | 42 | |
| **Sex** | | | | |
| Female, N (%) | 25 (69.4%) | 10 (63%) | 17 (40.5%) | *A vs. C, 0.039* |
| **Age** | | | | |
| Median (range) | 69.5 (38–83) | 64.5 (46–81) | 71 (66–74) | §*ns* |
| **BMI (kg/m²)** | | | | |
| Mean (SD) | 25.1 (5.1) | 26.3 (4.5) | 24.9 (2.4) | $*ns* |
| **Hypertension** | | | | |
| Yes | 25 (69.4%) | 7 (43.8%) | 16 (38.1%) | *A vs. C, 0.021* |
| No | 11 (30.6%) | 9 (56.2%) | 23 (54.7%) | |
| Unknown | 0 | 0 | 3 (7.1%) | |
| **Comorbidity Index*** | | | | |
| Median (range) | 1 (0–4) | 1 (0–4) | 0 (0–4) | §*A vs. C, 0.02* |
| No comorbidities (CCI = 0) | 7 (19.4%) | 6 (37.5%) | 22 (52.4%) | *A vs. C, 0.013* |
| Low burden (CCI ≤ 2) | 18 (50%) | 8 (50%) | 16 (38.1%) | *ns* |
| Moderate to high (CCI>2) | 11 (30.6%) | 2 (12.5%) | 4 (9.5%) | *ns* |
| **Blood test** | | | | |
| Leukocyte (×10⁹/L), mean (SD) | 5.9 (1.6) | 6.8 (1.7) | 6.4 (1.1) | $*ns* |
| Hematocrit (%), mean (SD) | 40 (3) | 41 (4) | 43 (3) | $*A vs. C, <0.001*<br>*B vs. C, 0.015*<br>*A vs. B, ns* |
| Thrombocytes count (×10⁹/L)<br>Median (range) | 332 (161–791) | 502 (162–903) | 227 (125–355) | §*A vs. B, <0.01*<br>*A vs. C, <0.001*<br>*B vs. C, <0.001* |
| eGFR, mean (SD) | 81.1 (15.2) | 82.3 (15.8) | 73.1 (13.2) | $*ns* |
| **Smoking** | | | | |
| Current smoker (%) | 4 (11.1 %) | 1 (6.25%) | 1 (2.4%) | *ns* |
| Former smoker (%) | 19 (52.8%) | 6 (37.5%) | 13 (31%) | *ns* |
| Never smoker (%) | 12 (33.3%) | 9 (56.3%) | 26 (61.9%) | *A vs. C, 0.042* |
| Unknown (%) | 1 (2.8%) | 0 | 2 (4.8%) | |

*Comorbidity scores were calculated using Charlson comorbidity index (CCI)

§Pairwise Wilcoxon rank-sum test

$ANOVA with post-hoc Tukey HSD test

Abbreviations: ns=not significant, A= *JAK2V617F*-positive patients with ET, B= *JAK2V617F*-negative patients with ET, C= healthy controls.

Figure S1

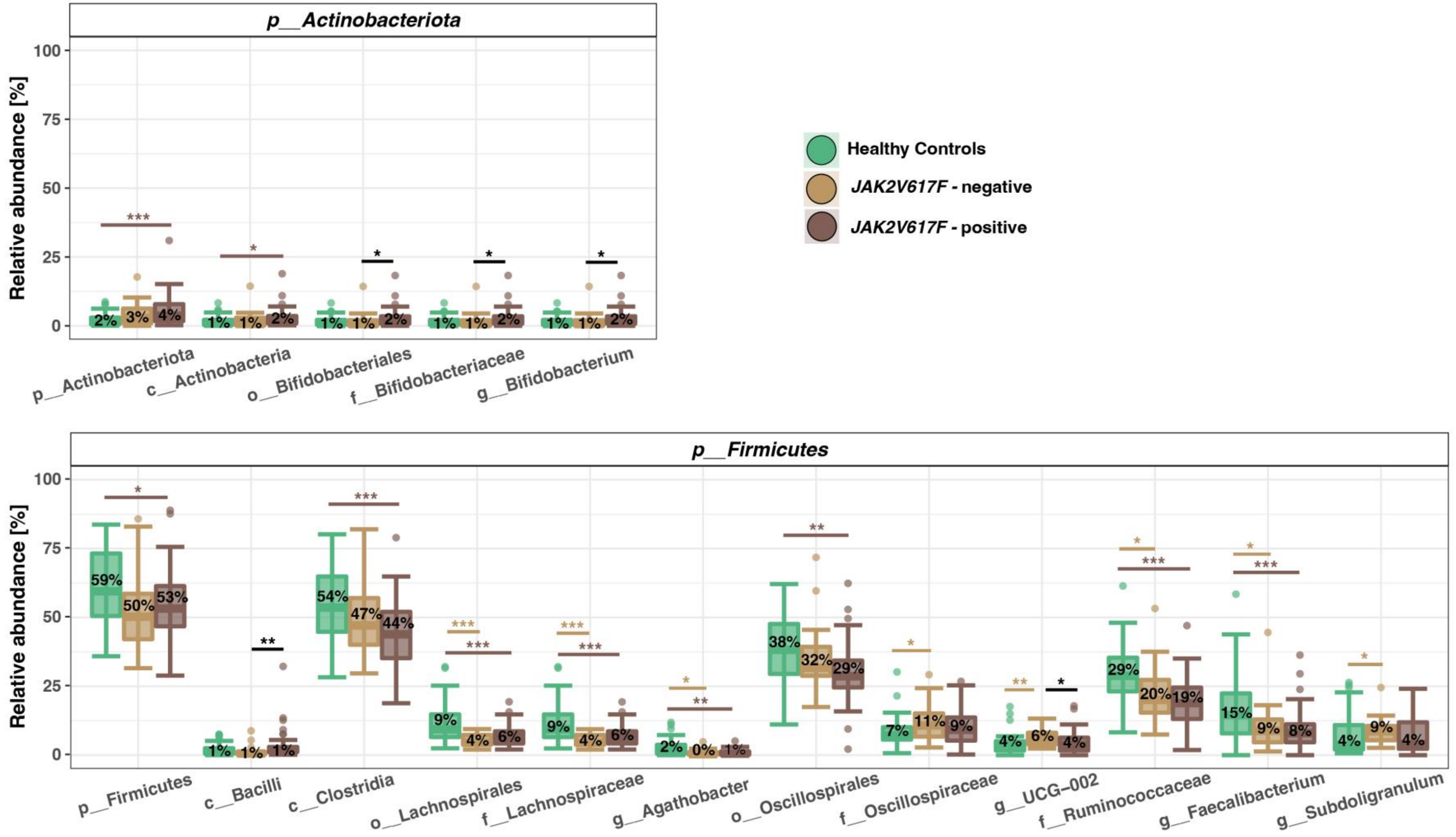
p__Actinobacteriota
Relative abundance [%]
100
75
50
25
0
2%
3%
4%
1%
1%
2%
p__Actinobacteriota
c__Actinobacteria
o__Bifidobacteriales
f__Bifidobacteriaceae
g__Bifidobacterium
Healthy Controls
JAK2V617F - negative
JAK2V617F - positive
p__Firmicutes
59%
50%
53%
54%
47%
44%
9%
4%
6%
2%
0%
38%
32%
29%
7%
11%
29%
20%
19%
15%
8%
p__Firmicutes
c__Bacilli
c__Clostridia
o__Lachnospirales
f__Lachnospiraceae
g__Agathobacter
o__Oscillospirales
f__Oscillospiraceae
g__UCG−002
f__Ruminococcaceae
g__Faecalibacterium
g__Subdoligranulum

**Figure S1. Differential abundance analysis of the gut microbiota in patients with ET according to mutations status compared with healthy controls**. Linear discriminant analysis Effect Size (LEfSe) was used to identify significantly different taxa between the groups. All taxa with an overall median proportion >1% and a LDA score >0.005 are shown. On the top taxa within the phylum *Actinobacteriota* are shown and in the bottom taxa within the phylum *Firmicutes are shown.*

Abbreviations: p, phylum; c, class; o, order; f, family; g, genus. Asterisks indicate the following levels of significance: *, p<0.05; **, p<0.01; and ***, p<0.001.
